# Comparative in vitro effectiveness of a novel contact lens multipurpose solution on *Acanthamoeba castellanii*

**DOI:** 10.1186/s12348-018-0161-8

**Published:** 2018-10-24

**Authors:** Alyssa C. Fears, Rebecca C. Metzinger, Stephanie Z. Killeen, Robert S. Reimers, Chad J. Roy

**Affiliations:** 1 Department of Microbiology and Immunology, Tulane School of Medicine, New Orleans, LA 70102 USA; 2 Department of Ophthalmology, Tulane University School of Medicine, New Orleans, LA 70102 USA; 3 Division of Microbiology, Tulane National Primate Research Center, Covington, LA 70433 USA; 4 Department of Environmental Health Sciences, Tulane School of Public Health and Tropical Medicine, New Orleans, LA 70102 USA

**Keywords:** *Acanthamoeba* keratitis, BioTrue, Opti Free, Alamar blue, In vitro effectiveness

## Abstract

**Background:**

A multipurpose contact lens cleaning solution (MPS) containing novel active ingredients under development was compared to two commercially available MPS solutions for effectiveness against *Acanthamoeba* isolates.

**Methods:**

The *Acanthamoeba* isolate *A*. *castellanii* was propagated for trophozoite or cyst-containing cultures for the purpose of assessment of effectiveness of each MPS. An alamar blue-based cellular respiration assay was used to assess effectiveness against trophozoites; Trypan blue hemocytometer-based microscopic counts measured cysticidal effects. To assess the general antimicrobial potency of each solution as controls for the anti-amoebic assays, comparative bactericidal effectiveness using *Serratia marcenses* was also performed.

**Results:**

Minimal effectiveness against either *Acanthamoeba* form was observed from either commercial MPS. In contrast, the novel MPS achieved complete kill within 1 h contact time for both *Acanthamoeba* trophozoite and cysts. Each commercial MPS required 6 h contact time to achieve a two to three log reduction in *S*. *marcenses*. In contrast, the experimental MPS achieved disinfection in 60 min contact time, and complete kill (< 1 CFU) at 90 min.

**Conclusions:**

Results suggest that the inclusion of a novel ingredient combination within the MPS under development clearly is required and is ideal for rapid and effective killing of *Acanthamoeba* species in the context of contact lens disinfection systems. The representative commercially available MPS used in this testing provided minimal effectiveness against the protozoa regardless of contact time. In addition, comparative results with the bacterial agent in the control study show distinct differences in the speed to disinfection with the novel MPS. Future MPS development should consider inclusion of novel chemical entities that are effective against *Acanthamoeba* species to speed disinfection and further reduce the exposure potential of users of contact lenses and cleaning systems.

## Background

*Acanthamoeba* are saprophytic infectious multiform protozoa found globally in numerous environments including waterways, swimming pools, distilled water, air samples, and even human tissues. *Acanthamoeba* exists in two forms including a metabolically active and more susceptible trophozoite form, and a dormant, highly resistant cyst form. It is also well-accepted that *Acanthamoeba* can act as a facultative parasite that is, among other pathologies, associated with sight-threating eye infection [1, 2]. The etiology of *Acanthamoeba* keratitis has been associated with contact lens use where the protozoa is transmitted from water in a contaminated storage case to the eye via the contact lens [3, 4]. Contact lens wear is a risk factor for contracting the disease, and incidence of *Acanthamoeba*-associated keratitis has increased worldwide over the past 20 years due to the popularity of contact lenses and the individuals that use reusable contact lenses [5–7].

Contact lens storage cases, therefore, represent a major exposure pathway for this pathogen into the eye of contact lens wearers. Current commercially available lens care multipurpose disinfection solutions (MPS) products have shown to provide minimal efficacy against *Acanthamoeba* species during routine cleaning and while soaking in contact lens cases [8–20]. The overall ineffectiveness of leading commercial MPS formulations against *Acanthamoeba* is not surprising as the majority of these products contain chemically similar active ingredients which possess minimal antiprotozoal properties. Specific MPS formulations have been implicated in the past as a risk factor for contracting *Acanthamoeba*-associated keratitis, with evidentiary ribosomal presence of the organism in the solution which indicated a clear relationship between use and disease [21]. The presence of *Acanthamoeba* spp. in contact lens cases punctuates the clear need for inclusion of novel active ingredients in newly formulated MPS.

A pH-balanced isotonic formulation comprised of novel antimicrobial active ingredients was specifically developed to address the presence of this protozoa, ultimately to lower the potential for infection. A MPS formulation that is biocidal and effective in eliminating all forms of the organism would greatly reduce the potential for encystment, thereby reducing the risk of MPS formulations harboring any *Acanthamoeba* species for any period of time, and eliminating the exposure pathway [17]. The aim of the present study was to assess the performance of a novel MPS with an experimental disinfection system against an indicator organism, and to assess the comparative evaluation of in vitro efficacy to two currently marketed contact lens disinfection solutions (BioTrue and Opti Free).

## Methods

### *Acanthamoeba castellanii* acquisition and propagation

*A*. *castellanii* (ATCC® 50,370™) derived from human eye infection scrapings was acquired from ATCC (Manassas, VA). Trophozoites were propagated at 25 °C (room temperature) in yeast extract peptone dextrose (YPD) media augmented with 1% penicillin, streptomycin, and amphotericin B. Cultures were gently swirled for 96 h at 25 °C, and concentrations were quantified and verified using an automated coulter cell counter (Vi-Cell XR, Beckman Coulter Instruments, Indianapolis, IN) and diluted to 10^4^ cell/mL for use. In separate cocultures, cysts were induced via 72-h incubation of 100 μL 10^6^ cells/mL in 10 mL encystment media (95 mM NaCl, 5 mM KCl, 8 mM MgSO_4_, 0.4 mM CaCl_2_, 1 mM NaHCO_3_, 20 mM Tris-HCl: pH = 9.0). Cysts were washed twice via centrifugation 5 min at 1000 rpm, enumerated using similar automated cell counting technique (Coulter), and then diluted with PBS to 10^5^ cells/mL for use.

### Trophozoites

Activity against the trophozoite form was evaluated via an Alamar Blue-based cellular respiration quantitative assay [22] and compared to currently marketed contact lens MPS Opti Free® (Alcon, Fort Worth, TX), BioTrue® (Bausch + Lomb, Bridgewater, NJ) or the newly developed solution ASP-57 (Asepticys, Alexandria, VA). Exactly 100 μL of media containing 10^5^ cells/mL *Acanthamoeba castellanii* cultures were commixed with 100 μL of either commercial or novel MPS solution with contact times ranging from 1 to 96 h before centrifugation for 10 min at 1000 rpm. The media-solution combinations were then aspirated, and 100 μL of fresh media added. Then, 10 μL of Alamar Blue (Reliablue™ Cell Viability Reagent (ATCC® 30-1014™, ATCC, Manassas, VA) was added and incubated. Absorbance was read after incubation at 570 nm via spectrophotometer. For statistical analysis of absorbance readings, unpaired *T* tests followed by *F* test to compare variances were performed using GraphPad Prism version 7.00 for Windows, GraphPad Software, La Jolla California USA.

### Cysts

Activity against *Acanthamoeba* cysts was determined via microscopic examination using Trypan blue to visualize cysts after treatment. Cysts were washed twice via centrifugation for 5 min at 1000 rpm, and diluted with PBS to 10^5^ cells/mL. Cysts and MPS formulations were each incubated at room temperature at a 1:1 ratio (10 μL:10 μL) using a duration of contact times (15, 30, 60, and 90 min). Thereafter, Trypan blue (10 μL) was used to visualize microscopically using a hemocytometer with a count of at least nine fields within the grid.

### Antimicrobial potency

The bactericidal effectiveness of each solution was determined against *Serratia marcescens* (ATCC 13880) using assay methods described in ISO 14729 Stand-Alone Test criteria which requires that the solution must be capable of reducing bacterial viability by three logs (99.9%) within a particular timeframe. Briefly, *S*. *marcescens* was propagated on sheep’s blood agar (SBA) and were harvested using methods described in the ISO 14729 standard, and adjusted through centrifugation and dilution with PBS to a final concentration of 2.0 × 10^5^ CFU/mL. Exactly 100 μL of each microbial suspension (CFU) was added to 9.9 mL for each solution in polypropylene tubes under sterile condition. Mixtures were incubated at 25 °C for 60, 90, or 360 min. Thereafter, 1 mL of each mixture was diluted with a neutralizing broth (Gibco, Detroit, MI) and streaked onto SBA, where it was incubated at 32 °C for 48 h. Plates were enumerated using the spread plate method, and the log reductions calculated. All assays were performed in triplicate.

### Test solutions

The multipurpose contact lens disinfection solutions BioTrue® (Bausch + Lomb, Bridgewater, NJ) and OptiFree® puremoist (Alcon, Fort Worth, TX) were acquired from commercial sources. The experimental MPS formulation, ASP-57, was obtained from Asepticys (Asepticys LLC, Alexandria, VA). The active ingredients of each solution tested are detailed in Table 1.

**Table 1:** Active ingredients of commercial MPS formulations and corresponding minimum time for disinfection based upon manufacturer’s instructions for use. No minimum disinfection time is listed for ASP-57 as this care solution is a research and development product and has not yet been approved for use by the US FDA

MPS	Active ingredients	Conc. (*w*/*w*)	Min. time for disinfection (h)
ASP-57	Stabilized Cl-O_2_ (as sodium chlorite)	0.02%	–
	Alkyl(ethylbenzyl)dimethylammonium chloride	0.0001%	
	Ammonium chloride	0.025%	
BioTrue	Polyaminopropyl biguanide	0.00013%	4
	Polyquaternium	0.0001%	
Opti Free	Myristamidopropyl dimethylamine	0.0005%	6
	Polyquaternium-1	0.001%	

## Results

### Neither of the commercial MPS tested killed *Acanthamoeba* trophozoites

**Fig. 1:**
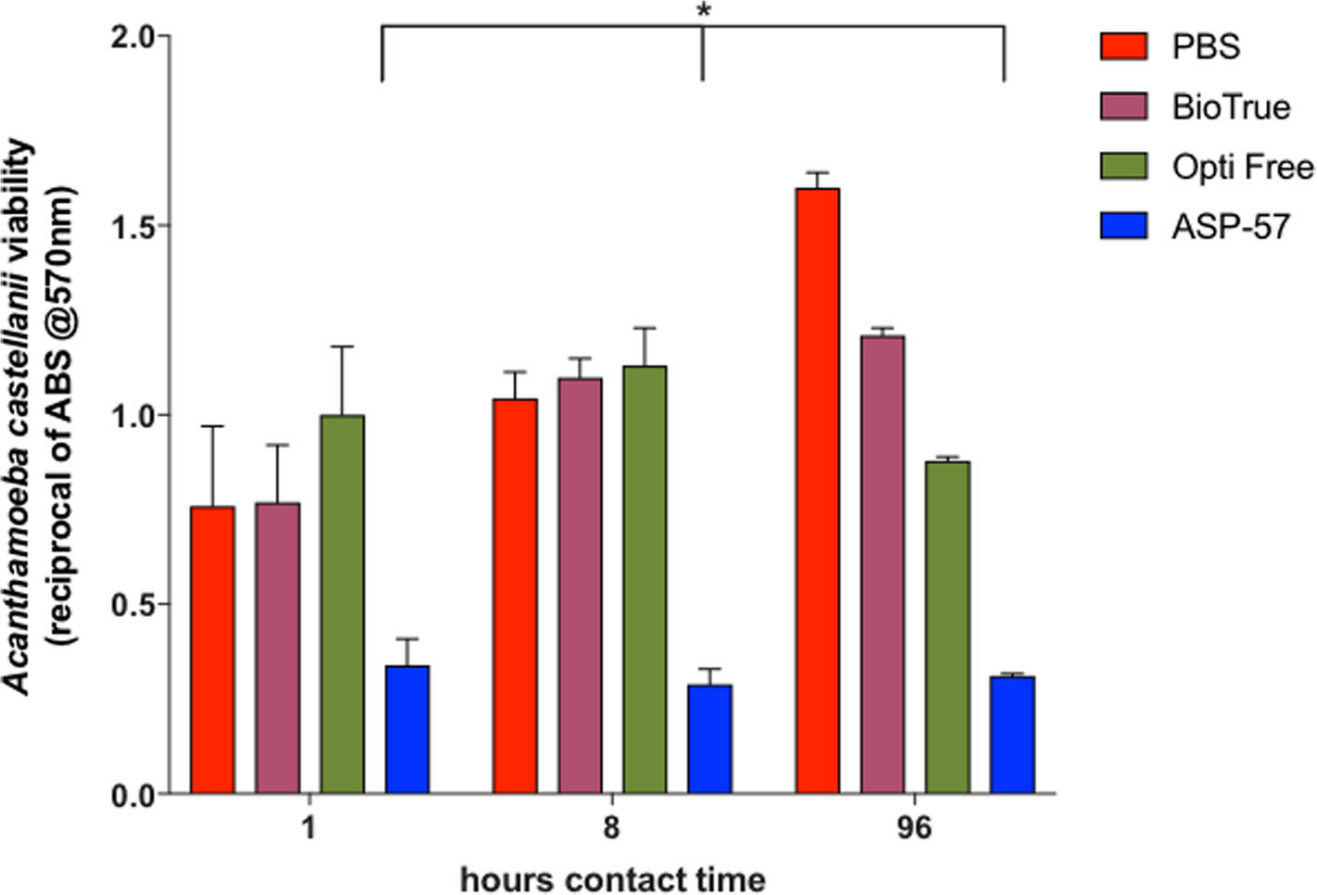
The efficacy of various contact lens care solutions against *A*. *castellanii* trophozoites using an alamar-blue colorimetric assay. *A*. *castellanii* trophozoites (3.25 × 10^4^ cells/well) were incubated with each MPS solution with contact times ranging from 1 to 96 h, aspirated, and then after the addition of fresh media, alamar blue was added and the solution incubated; absorbance was read after incubation. Statistical significance at *p* > 0.01 indicated by asterisk comparing ASP-57 v. control (PBS) at all contact times attempted

The alamar blue quantitative assay and protocol previously described [22] was initially confirmed and optimized within our laboratories prior to testing to ensure consistency within this particular test system, which included identification of the number of trophozoites to be used per well (3.25 × 10^4^ cells/well) to ensure proper reaction with the alamar blue reagent. In addition, the reciprocal of the absorbance values (570 nm) were used because the decrease of signal directly correlates to trophozoite viability and cellular respiration of the colorimetric reagent. Three contact times (1, 8, and 96 h) were used in separate assays. The results of the testing indicated (Fig. 1) that neither of the commercial solutions resulted in any substantial reduction in trophozoite viability at the 1 and 8 h timepoints, essentially matching that of PBS sham treatment. In contrast, the novel MPS ASP-57 resulted in significant reduction of trophozoites at the earliest and all other contact times attempted, indicating a complete kill subsequent to a 1 h contact time. Extending contact time to 8 or 96 h did not appreciably improve the efficacy of either commercial MPS; however, a significant decrease was observed in the 96 h Opti Free solution when compared to PBS sham treatment.

### Neither of the commercial MPS tested killed *Acanthamoeba* cysts

**Fig. 2:**
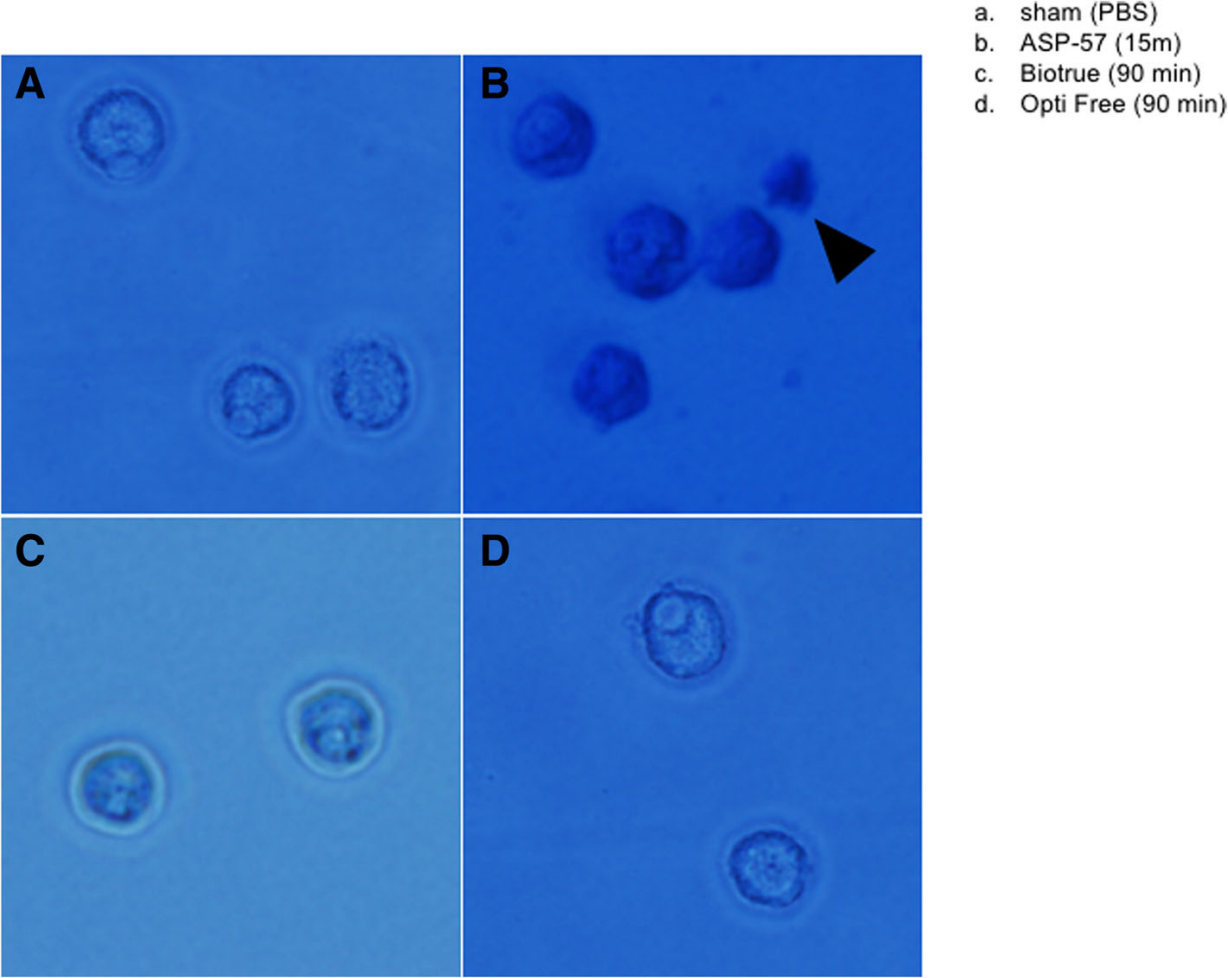
Photomicrographs of A. castellanii cysts commixed with various contact lens care solutions after staining with trypan blue. **a**. Sham (PBS) treatment. **b**. ASP-57 treatment after 15-min incubation. **c**. BioTrue treatment. **d**. Opti Free treatment. Arrows denote cellular debris from dead cyst (**b**.). All images at magnification × 400

Trypan blue is commonly used as a vital stain to selectively color dead cells blue, whereas live cells with intact cell membranes are not colored. The dye exclusion method has worked effectively for viability with *Acanthamoeba* in past studies [23] and we utilized this method for direct microscopic counting of viable cysts on a hemocytometer for the current study. A number of contact times with the solutions under testing were attempted (15, 30, 60, and 90 min) and at least two individuals within the laboratory participated in counting. Collective results of the microscopic counts (Table 2) showed cysticidal activity of ASP-57 MPS at the earliest timepoint (15 min), effectively killing > 70% of cysts counted in comparison to PBS sham treatment. Killing effectiveness increased at later timepoints, with ASP-57 killing essentially all cysts (30 min, 96%; 60 min, 97%; 90 min 100%). In contrast, neither of the commercial solutions achieved a killing efficiency more than 35% at any of the timepoints attempted, with the exception of one (Opti Free, 51%) at 90 min contact time. Qualitative analysis of the effect of the different solutions on *A. castellanii* cysts (Fig. 2) clearly demonstrates the deleterious effects of exposure to the novel MPS ASP-57 (Fig. 2b) when compared to healthy, sham-treated (PBS) cysts (Fig. 2a). The exclusion dye (trypan blue) has been internalized in the cysts commixed with ASP-57, indicating that the plasma membrane has been completely compromised, with cellular contents proximal to the dead cyst (Fig. 2b). In contrast, cysts commixed with either of the commercial MPS show little change when compared to sham-treated samples (Fig. 2c, d), which is confirmed quantitatively in our enumeration (Table 2).

**Table 2:** *Acanthamoeba castellanii* cyst viability counts after exposure to different MPS formulations. Cultures containing *A*. *castellanii* cysts were co-mixed for varying contact times, stained with Trypan blue, and then transferred to a hemocytometer for microscopic enumeration. A total of nine grids were counted; separate experiments were performed and quantitative analysis performed by two technicians blinded to the treatments. Counts reflect percentage dead cysts of total counted within the collective nine grids; numbers expressed as arithmetic mean ± standard deviation

MPS	Contact time (min)			
	15	30	60	90
PBS (sham)	–	–	–	–
ASP-57	72.8 ± 26.9	96.4 ± 5.1	97.5 ± 3.5	100 ± 0.0
BioTrue	5.6 ± 2.9	15.6 ± 22.1	24.2 ± 22.4	32.9 ± 4.9
Opti Free	23.2 ± 5.8	23.6 ± 2.0	19.9 ± 15.3	51.1 ± 7.9

### There are temporal differences in effectiveness of commercial MPS v. ASP-57 against *S*. *marcenses*

**Fig. 3:**
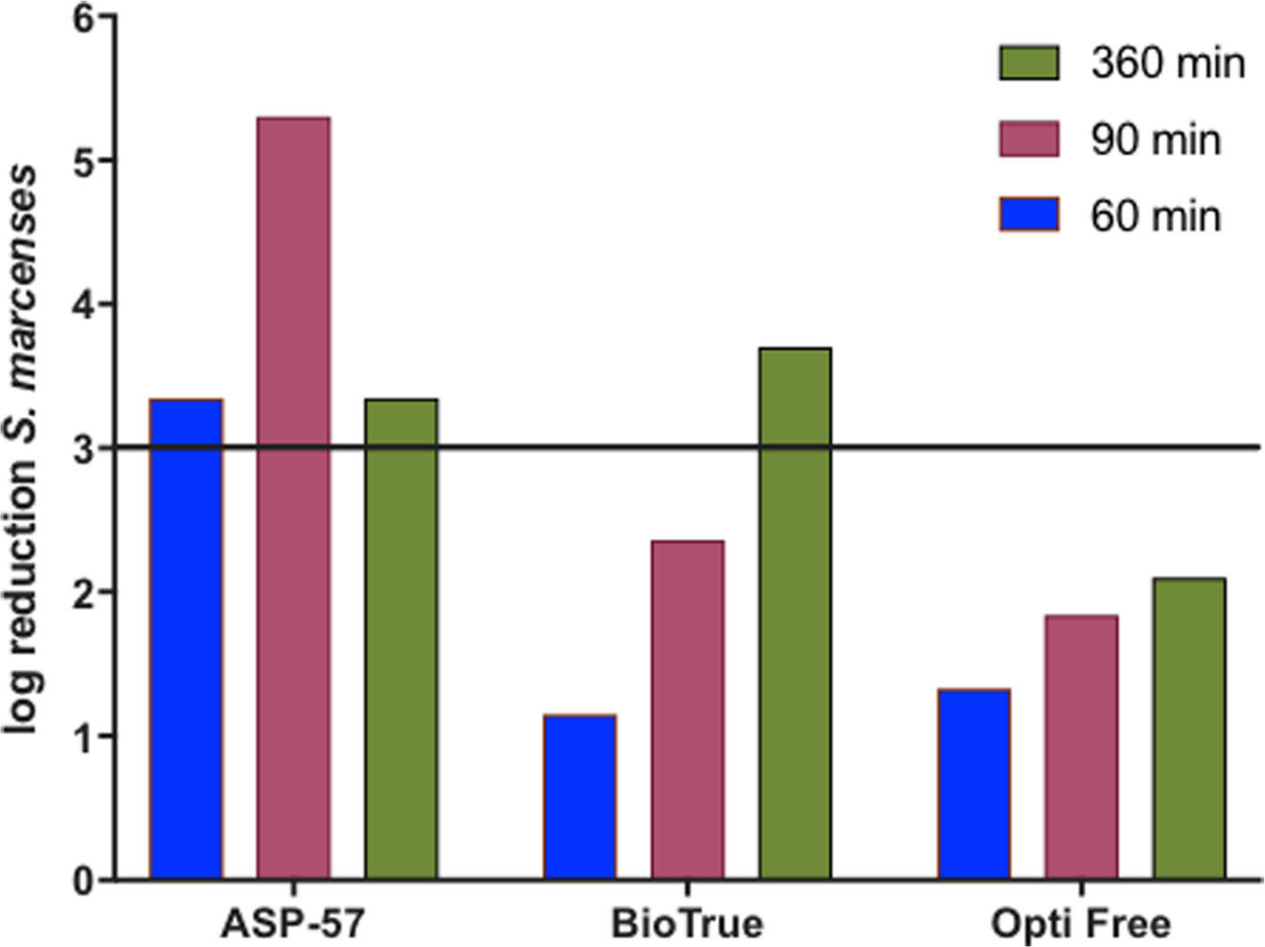
Bactericidal effectiveness of various contact lens care solutions against S. marcescens. A microbial suspension of *S. marcescens* was added to each solution and incubated at contact times noted. Each mixture was diluted, streaked onto plates, and enumerated for log reduction calculations. Three-log reduction threshold is denoted by the horizontal line from the x-axis

*Serratia marcescens* is one of five microorganisms included in the panel of agents required by ISO 14729 for testing the disinfection qualities of contact lens disinfection solutions. A three-log reduction capacity for the bacterial agents included in the panel at the minimum contact time of 4 h is considered consistent with acceptable disinfection capacity as ISO acceptance criteria. *S*. *marcescens* is considered the most resilient among the bacterial agents on the panel, and thus was chosen as an indicator organism for MPS potency control in the present study. The results of the testing (Fig. 3) indicate that the novel MPS ASP-57 achieved log reduction (> 5) at 60 min contact time compared to Biotrue (1.15) and Opti Free (1.33). ASP-57 produced a sterilizing effect (< 1 CFU; 5.30) at 90 min contact time; both commercial solutions failed to achieve minimum log reduction at this timepoint (Biotrue 2.36, Opti Free 1.84). Only after 6 h contact time did one of the two commercial solutions achieve the required 3-log reduction (Biotrue 3.70) while the other failed (Opti Free 2.10).

## Discussion

Development of contact lens care solutions that incorporate disinfection systems to properly address the rapid and complete killing of protozoal agents such as *A*. *castellanii* has clearly not been the focus of manufacturers for the past several years. This may be a consequence of no identifiable procedure or regulatory standard for determination of *Acanthamoeba* disinfection [24]. The purpose of the current study was to demonstrate the disinfection potential of a novel MPS (ASP-57) that uses a different disinfection system than most of the currently marketed care solutions and to compare with two of the leading commercial MPS formulations in the context of effectiveness against an indicator organism (*S*. *marcenses*) and performance against *A*. *castellanii*.

The potency of ASP-57 to kill *Acanthamoeba* trophozoites and cysts in this study’s timeframe is unusual. Comparator solutions failed to kill an appreciable number of *Acanthamoeba* and suggest that the protozoa could maintain viability for longer periods of time in contact lens holding cases. Prior study of MPS formulations to kill *Acanthamoeba* have indicated very low or no effectiveness for the commercially available solutions used as comparators in the current study [20, 25–29]. The inability of current MPS formulations to effectively kill this pathogen may be due to the choice and prevailing concentration of disinfecting agents in the majority of these formulations. Commercial MPS active ingredients contain suboptimal concentrations of biguanide salts (e.g., polyaminopropyl biguanide) or quaternary ammonium compounds (e.g., polyquaterium-1), usually in combination. The choice and suboptimal concentrations of active ingredients used in commercial formulations lead to poor to no efficacy against recalcitrant organisms such as *Acanthamoeba* as well as anemic performance against more commonly found bacterial agents such as *S*. *marcenses*. The recalcitrance of *Acanthamoeba* was punctuated by testing the kill potential of other common antiseptics such as chlorhexidine and povidone-iodine, both of which proved only marginally effective against trophozoites and cysts (data not shown).

The use of novel active ingredients in our experimental care solution ASP-57 resulted in swift and robust disinfection, as initially evidenced by the *S*. *marcescens* control assay performed. The full biocidal panel as dictated by ISO 14729 was performed with ASP-57 in addition to the single agent assay with similar log reduction and timing as our experience with *S*. *marcescens* (data not shown). The remarkable performance of this novel disinfection system against the bacterial and fungal panel in the preliminary biocidal assay was prophetic when considering the potential potency against the protozoal agent *A*. *castellanii*. The results of the *A*. *castellanii* trophozoite viability colorimetric-based assay clearly showed a distinct and statistically significant difference in the performance of ASP-57 when compared to the active ingredient combinations used in the commercial comparator care solutions. The performance of the comparator solutions against both *A*. *castellanii* forms (trophozoites and cysts) was not surprising as similar results have been demonstrated in past studies using a variety of laboratory methods for enumeration and qualitative analysis [20, 25, 26]. Similarly, there have been very few (one) marketed, now discontinued MPS product with any chlorine-based disinfection componentry, and its effectiveness against *Acanthamoeba* sp. was not reported [30]. Although our study used a genotypic strain of *A*. *castellanii* to assess initial performance, it was limited to a single strain rather than inclusion of multiple strains, including the T4 genotype [25, 26]. We recently have propagated *Acanthamoeba polyphaga* and used similar testing methodologies as were used with our *A*. *castellanii* study which yielded surprisingly similar results (data not shown).

## Conclusions

This study suggests that an alternative approach to active ingredient composition should be considered when pathogens such as *Acanthamoeba* are of concern to users of reusable contact lens. It is clear from the current study and numerous past studies that the selected disinfection systems, including polyquaterium/biguanide(PHMB) or polyquaterium/myristamidopropyl dimethylamine systems are not ideal for rapid or effective killing of protozoal species of concern such as *A*. *castellanii*. The current study is limited in scope and interpretation by the use of only two MPS formulations for a comparator to ASP-57; future studies will expand our scope of alternative MPS solutions, including multipart H_2_O_2_-based disinfection systems, to provide a more comprehensive assessment of commercially marketed products against *Acanthamoeba*. Next-generation disinfection systems for contact lens care solutions should consider the benefits of incorporation of novel agents such as those within the experimental solution ASP-57 to ensure rapid and complete disinfection of protozoal and other commonly encountered microbial contaminants.
